# Beyond Focal Pests: Impact of a Neonicotinoid Seed Treatment and Resistant Soybean Lines on a Non-Target Arthropod

**DOI:** 10.3390/insects7040064

**Published:** 2016-11-11

**Authors:** Tülin Özsisli, Deirdre A. Prischmann-Voldseth

**Affiliations:** 1 Department of Plant Protection, Agricultural Faculty, Kahramanmaraş Sütçü İmam University, Avşar Campus, Kahramanmaraş 46040, Turkey; tulin@ksu.edu.tr; 2 Department of Entomology 7650, North Dakota State University, PO Pox 6050, Fargo, ND 58108, USA

**Keywords:** thiamethoxam, host plant resistance, *Tetranychus urticae*, *Ambylseius fallacis*

## Abstract

Integrated pest management (IPM) tactics may effectively control focal pests, but it is also important to test the compatibility of different tactics, and consider non-target organisms. We investigated the effects of a neonicotinoid seed treatment and *Rag* resistance genes used for soybean aphid (*Aphis glycines* Matsumura) control on reproduction of a non-target herbivore (twospotted spider mite, *Tetranychus urticae* Koch) in short-term greenhouse experiments. We also examined interactions between spider mites and a specialist phytoseiid mite [*Ambylseius fallacis* (Garman)] and assessed the effects of a co-occurring opportunistic omnivore [*Frankliniella occidentalis* (Pergande)] by including thrips density as a covariate. There were no interactive or main effects of the presence of *Rag* genes on the densities of any of the arthropods. Overall, effects of the seed treatment on spider mite densities varied, with no difference when mites were confined in clip cages, and higher populations on seed-treated plants when on whole plants. Predatory mites had a consistent negative impact on spider mites, and densities of *A. fallacis* immatures were similar between seed treated and non-seed treated plants. However, the relationship between spider mite and thrips densities was different between these two plant types, but only in the clip cage experiment lacking predatory mites. This research highlights the importance of considering how IPM tactics might affect non-target organisms.

## 1. Introduction

When attempting to manage arthropods, even within simplified monocultures, management practices targeting one pest can have unforeseen and undesirable consequences on non-target arthropods. Using multiple control tactics to combat pests is one of the goals of integrated pest management (IPM) programs, and ideally would lead to synergistic pest control. Conversely, control tactics may not be complementary. Well-known examples include non-selective insecticides and resistant host plants interfering with biological control [1,2]. It is also important to consider how pest management tactics impact non-target herbivores, which could replace target pests and injure host plants [3,4], or serve a positive role as alternative prey for natural enemies [5]. Obtaining basic information on how relevant organisms are affected by the use of individual and combined management tactics can help develop successful IPM programs.

Soybean aphids (Hemiptera: Aphididae, *Aphis glycines* Matsumura) are invasive pests native to China that have created considerable issues for producers since their discovery in the United States [6], with one study estimating the economic cost of uncontrolled soybean aphid populations to be around 700 million US dollars [7]. These insects ingest phloem sap, and their feeding can reduce the quantity and quality of soybean (*Glycine max* L.) yields [8,9]. Foliar insecticides are one of the primary methods used to manage soybean aphid populations [6], although the use of systemic neonicotinoids applied via seed treatments has been increasing [10,11]. When applied as a seed treatment, these systemic insecticides move from the roots to plant tissues via the xylem, and are effective against a wide array of sucking insects, including aphids and thrips [12,13,14].

There has also been interest in using host plant resistance to manage soybean aphids, particularly using soybean varieties that possess *Rag* (Resistance to *Aphis glycines*) genes [15,16]. Several *Rag* genes have been characterized, with the presence of the genes primarily conferring antixenosis and/or antibiosis [16]. Because soybean aphid biotypes have been found that can successfully colonize and develop on soybeans with *Rag* genes [17,18], researchers have been investigating pyramided resistance, in which multiple *Rag* genes are present within the same plant [19,20,21]. There is some information that the presence of *Rag* genes can impact the behavior and biology of other herbivores associated with soybean [22,23].

Several other herbivorous arthropods feed on soybean, including spider mites (Acari: Tetranychidae) [24]. Spider mites tend to be most problematic when environmental conditions are hot and dry [25,26]. They feed by piercing leaf cells with stylet-like chelicerae and sucking up the contents [27]. In the Midwestern United States, spider mites are considered occasional pests of soybean [24], although they do negatively affect soybean plants, including reducing chlorophyll content of leaves [28], photosynthesis [29], dry weight of above-ground plant material [30], and seed weight [30,31]. Thus, it is important to consider how soybean aphid management strategies might influence their populations.

Neonicotinoid seed treatments are known to contribute to higher spider mite populations [32,33,34,35,36,37] and can have negative impacts on several types of natural enemies [34,38,39,40]. Phytoseiid mites (Acari: Phytoseiidae) are important predators of spider mites in many agroecosystems [41]. Neonicotinoids are not always toxic to phytoseiid mites [42,43,44], although this likely depends on the specific chemical, dosage, method of exposure, mite species, and life stage. In some situations, phytoseiid mites exposed to neonicotinoids had higher levels of mortality [38,42,45], reduced oviposition rates [42,44,45], and reduced prey consumption [43].

As interest in using soybean varieties with *Rag* genes grows, it is important to understand the potential consequences of their use for non-target soybean arthropods, as well as any interactive effects with commonly-used neonicotinoid seed treatments. The goal of this study was to examine the effects of these two control methods for soybean aphids on short-term reproduction of a non-target herbivore, the twospotted spider mite (*Tetranychus urticae* Koch). We also investigated the effects of the seed treatment and *Rag* genes on spider mite control by a predatory phytoseiid mite [*Ambylseius* (*Neoseiulus*) *fallacis* (Garman)]. Although herbivorous western flower thrips [Thysanoptera: Thripidae, *Frankliniella occidentalis* (Pergande)] was not an a priori target of these experiments, they were present in the environment and thus we used the densities of this co-occurring opportunistic omnivore as a covariate in our analyses, as they are known to consume spider mites in addition to plant tissue [46]. Our expectation was that predator densities would be lower, and densities of spider mites higher on soybeans with the seed treatment, but that there would be no impact of the *Rag* genes, either alone or in combination with the seed treatment.

## 2. Materials and Methods

All experiments were a completely randomized design with a full factorial treatment arrangement. There were two levels of an insecticidal neonicotinoid seed treatment (untreated and treated) and four near-isogenic soybean lines: (1) control: soybeans with no *Rag* resistance genes that are susceptible to soybean aphids; (2) Rag1: soybeans with the *Rag1* soybean aphid resistance gene; (3) Rag2: soybeans with the *Rag2* soybean aphid resistance gene; and (4) Rag1 + Rag2: soybeans with both the *Rag1* and *Rag2* soybean aphid resistance genes. A second clip-cage experiment included an additional treatment level in addition to the seed treatment and soybean line, i.e., with and without the predatory mite *Amblyseius fallacis*.

Information on the parentage and development of these lines is provided in [20,21]. The seed used in these experiments was from the same lot as was used in a multi-location field study, where soybean aphid densities were significantly lower on lines with the *Rag* genes [21]. The insecticidal seed treatment was a neonicotinoid insecticide (thiamethoxam, Cruiser 5FS, Syngenta Crop Protection, Inc., Greensboro, NC, USA; lowest label rate for soybean of 0.0756 mg AI per seed = 50.0 g AI per 100 kg seed).

Plants were grown and experiments were conducted in a greenhouse (25 ± 4 °C, 40%–75% RH, 16L:8D). Three to four seeds were planted in each pot (10.5 cm × 10.5 cm × 12.5 cm high) in Sunshine Mix #1 growing mix (Sun Gro Horticulture, Agawan, MA, USA) and thinned to one plant per pot immediate prior to experiments. Soybean seedlings were placed in cages with thrips proof mesh (BD44545F BugDorm, MegaView Science Co., Ltd., Taichung, Taiwan) at the VC (cotyledon) growth stage.

Spider mites used in experiments were *Tetranychus urticae* and were collected from a greenhouse and maintained on soybean (RG607RR, Roughrider Genetics^®^, NDSU Research Foundation, Fargo, ND, USA), starting in 2011 at 21 ± 2 °C, 40%–65% RH, and 16L:8D. Spider mites were transferred to experimental plants on detached leaf pieces. For each experiment, three adult female spider mites were transferred using a small paintbrush to a soybean leaf piece ringed with wet tissue resting on wet cotton in a plastic cup (Solo^®^ P125, 36.9 mL, Solo Cup Company, Lake Forest, IL, USA). Cups were prepared 1 day prior to experiments, chilled at 4 ± 2 °C until needed, and transported to the greenhouse in coolers.

### 2.1. Clip Cage Experiment

Soybeans were planted on 18 June 2013 and were at the V1 growth stage (one fully expanded trifoliate) at the start of the experiment on 2–3 July 2013. One leaf piece (i.e., three adult female spider mites) was placed on the adaxial surface of a unifoliate leaf of each experimental plant. A clip cage (two 5.2 cm diameter × 1.0 cm high plastic cylinders, ringed with foam, with nylon organdy mesh covering the exterior sides) mounted on a hair clip was then placed over the unifoliate, leaving a gap so that the mites could move to the underside of the leaf. Clip cages were supported using 16 gauge wire and wooden stakes sunk into the soil. Pots were then placed inside mesh cages (61 cm × 61 cm × 91.4 cm, mesh with 330 holes per 2.54 cm^2^). Soybeans were watered as needed (approximately every 3 to 4 days with tap water), and were not fertilized. Eight days later (10–11 July 2013), infested leaves were detached and placed in individual plastic self-sealing bags that were chilled (4 ± 2 °C) until spider mites (adult females, larvae + nymphs, and eggs) were counted using a dissecting microscope. The effects of neonicotinoids on spider mite egg production have previously been observed six days after exposure [47]. Any thrips (adults + larvae) present on plants at the end of the experiment were also counted. There were 10 replicates of each treatment.

### 2.2. Whole Plant Experiment

Soybeans were planted on 3 July 2013 and the experiment was started on 17–18 July 2013. One leaf piece (i.e., three adult female spider mites) was placed on the adaxial surface of the first trifoliate. The entire plant was then caged with a mesh bag (BugDorm insect rearing bag, 100 cm long × 66 cm wide; MegaView Science Co., Ltd., Taichung, Taiwan) draped over two crisscrossed wires so that the bag was suspended over the foliage. Six days later (23–24 July 2013), the soybean growth stage and densities and location of all arthropods were counted using a dissecting microscope. There were 10 replicates of each treatment.

### 2.3. Clip Cage Predator Addition Experiment

Predatory mites were purchased from Buglogical Control Systems Inc. (Tucson, AZ, USA) and maintained on detached soybean leaf arenas. They were fed spider mites ad libitum every 3 to 4 days and colonies were renewed every 3 to 4 weeks. The experiment was started when the plants were at the V1 growth stage. One leaf piece (i.e., three adult female spider mites) was placed on the adaxial surface of the middle leaflet of the first trifoliate and covered by a clip cage, as described earlier. Three days after spider mites were added, 2 of the 10 replicates were destructively sampled and spider mite densities were quantified. The following day, one adult female *A. fallacis* was added to each clip cage of half of the remaining replicates (i.e., *n* = 4), while the remaining four replicates did not receive predators. Eight days after the spider mites were added to the experimental plants, the infested leaf with the clip cage was detached, placed in a labeled plastic bag and chilled (4 ± 2 °C) until the densities of all arthropods were counted using a dissecting microscope.

### 2.4. Statistics

Data were analyzed using SYSTAT^®^ (Systat Software Inc., San Jose, CA, USA) [48]. Histograms and Bartlett’s test were used to assess whether these data met assumptions necessary for parametric statistics and whether the data needed to be transformed. Data points from plants on which spider mites failed to establish after infestation (i.e., spider mite densities ≤10 at the end of the experiment), plants with defective clip cages, and severe outliers as identified by the statistical program were eliminated from analyses.

For the first and second experiment, data were analyzed using a general linear model, with spider mite density as the dependent variable (all life stages at the end of the experiment, mites per clip cage and mites per plant, respectively), and insecticidal seed treatment (ST) and soybean line (Line) as the categorical independent variables. For the second experiment we analyzed arthropod data from two different scales, namely plant-level spider mite density data (all life stages) and data from leaves where spider mites were present. For the clip cage experiment with *A. fallacis*, we first analyzed spider mite density data (mites per clip cage) immediately prior to the addition of predators (i.e., 3 days after cages were infested with spider mites) using general linear model (GLM) with two independent variables (ST, Line). We then analyzed data from the end of the experiment (i.e., 8 days after cages were infested with spider mites; all life stages at the end of the experiment) and included the presence/absence of *A. fallacis* (Predator) as an independent variable. Effects of ST and Line on the density of *A. fallacis* offspring (eggs plus immatures at the end of the experiment) were analyzed separately using GLM. We used general linear regression to explore relationships between the spider mite and *A. fallacis* densities (motile predator life stages only) on both seed treated and non-seed treated plants.

Thrips were not present on leaves to which spider mites were added, and the level of thrips damage appeared similar among plants. However, since this thrips species has been shown to eat spider mites in addition to plant material [46], we included thrips density (adult + larvae, square root X + 0.5 transformed) as a covariate (all main and interactive effects) in all general linear model (GLM) analyses. One exception was the 3 day assessment of the clip cage predator experiment, as few thrips were present at that time. If covariate × independent variable interactions were non-significant, they were removed from the model [49]. If covariate × independent variable interactions were significant, we used correlation analyses to explore the relationships between the densities of spider mites and thrips between different groups (e.g., non-seed treated versus seed treated plants), running separate analyses using the full data set and then using only the data from plants where thrips were present. Additionally, we also analyzed the effects of insecticidal seed treatment and soybean line on spider mite densities using only data from plants lacking thrips for the first clip cage experiment.

## 3. Results

### 3.1. Clip Cage Experiment

**Figure 1:**
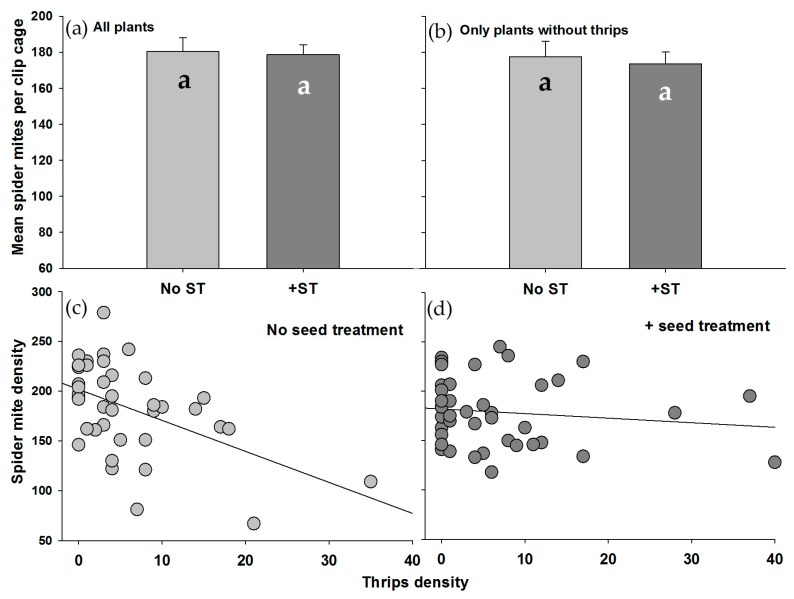
Mean arthropod density (±SE) per clip cage at the end of an 8 day greenhouse experiment on soybean: (**a**) Spider mites using data from all plants; (**b**) Spider mites using data only from plants lacking thrips. Relationship between the densities of spider mites and thrips within clip cages; (**c**) On soybean plants without a thiamethoxam seed treatment; (**d**) On soybean plants with a thiamethoxam seed treatment. Correlations were generated using data from all plants, although results were similar when data from plants lacking thrips were used.

The densities of spider mites at the end of the experiment were not affected by the identity of the soybean line, and there was no interactive effect of soybean line and insecticidal seed treatment (ST × Line, *df*_3,70_, *F* = 0.437, *p* = 0.727; Line, *df*_3,70_, *F* = 1.967, *p* = 0.127). This did not change when only using data from plants lacking thrips (ST × Line, *df*_3,50_, *F* = 0.065, *p* = 0.978; Line, *df*_3,50_, *F* = 1.445, *p* = 0.241). Even though no thrips were present within clip cages when spider mites were added, at the end of the experiment thrips were present on 72.5% of plants (mean per cage ± SEM = 6.4 ± 1.0, no ST = 5.8 ± 1.2, +ST = 7.0 ± 1.5). There was a significant seed treatment by covariate interaction (ST × Thrips, *df*_1,64_, *F* = 1.001, *p* = 0.032), indicating that the effect of the insecticidal seed treatment on spider mite densities depended on thrips densities. There was a significant negative association between the densities of spider mite and thrips, but only on soybeans lacking the seed treatment (Figure 1; no ST: Bartlett Chi-square statistic = 6.280, *p* = 0.012; +ST: Bartlett Chi-square statistic = 0.766, *p* = 0.385). These relationships were similar even when data from plants lacking thrips were omitted from the analyses (no ST: Bartlett Chi-square statistic = 7.091, *p* = 0.008; +ST: Chi-square statistic = 0.042, *p* = 0.837). When only using data from thrips-free plants, there was no impact of the seed treatment on spider mite densities (Figure 1; ST, *df*_1,50_, *F* = 0.165, *p* = 0.687).

### 3.2. Whole Plant Experiment

**Figure 2:**
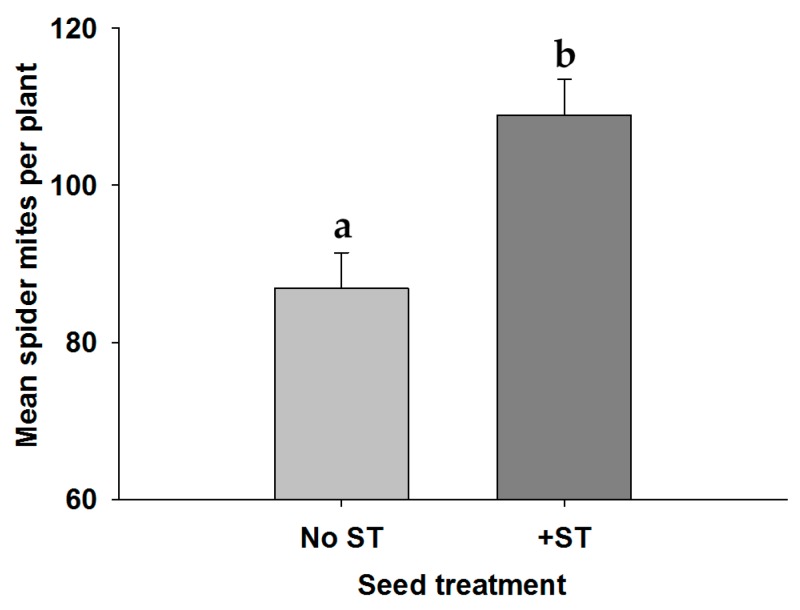
Mean density of spider mites per plant (±SE) at the end of a 6 day greenhouse whole plant experiment on soybean.

We only recorded the trifoliate, not the specific leaflet, where spider mites were initially infested, but at the end of the experiment, most spider mites were still on the trifoliate on which they had been placed (92.5% of plants). At a whole plant level, similar to the clip cage experiment, the main and interactive effects of soybean variety on spider mite densities were not significant (ST × Line, *df*_3,69_, *F* = 0.480, *p* = 0.697; Line, *df*_3,69_, *F* = 0.343, *p* = 0.794). Spider mite densities (all life stages) were higher on seed treated plants (Figure 2; ST, *df*_1,69_, *F* = 10.879, *p* = 0.002).

Thrips were present on all experimental plants at the end of the experiment (mean per infested plant ± SEM = 21.3 ± 1.3, no ST = 19.7 ± 2.0, +ST = 23.1 ± 1.8; mean per spider-mite infested leaf ± SEM: no ST = 6.5 ± 1.0, +ST = 3.6 ± 0.6). However, there was consistently no effect of thrips density on spider mite density, either when considering thrips at the whole-plant level (main and all interactive effects of Thrips, *p* ≥ 0.240) or thrips densities only on spider-mite infested leaves (main and all interactive effects of Thrips, *p* ≥ 0.322). However, most of the thrips population was associated with newer plant growth (no ST = 67.6% ± 3.7%, +ST = 86.2% ± 1.8%) rather than spider-mite infested leaves.

### 3.3. Clip Cage Predator Addition Experiment

Three days after spider mites were added to clip cages, no spider mite eggs had hatched, and only three samples (all non-seed treated plants) had thrips (mean per infested cage ± SEM = 2.7 ± 0.9). There was no impact of soybean line (control = 71.5 ± 2.6, Rag1 = 71.8 ± 4.5, Rag2 = 79.8 ± 3.1, Rag1 + Rag2 = 62.3 ± 7.6) or seed treatment (no ST = 74.8 ± 2.8, +ST = 67.9 ± 4.5) on spider mite densities at this time (ST × Line, *df*_3,8_, *F* = 0.635, *p* = 0.613; ST, *df*_1,8_, *F* = 1.984, *p* = 0.197; Line, *df*_3,8_, *F* = 2.148, *p* = 0.172).

At the end of the experiment (8 days after spider mite infestation), thrips had colonized 65.5% of plants with a mean density (±SE) of 6.5 ± 1.1 per infested cage, although they did not have a significant impact on spider mite densities (main and all interactive effects of Thrips, *p* ≥ 0.172), and so the covariate was dropped from the model. Spider mite densities were consistently lower in the presence of *A. fallacis* (no predator = 201.5 ± 7.9, +predator = 147.2 ± 11.2), although they were unaffected by soybean line (control = 160.6 ± 17.5, Rag1 = 172.5 ± 16.9, Rag2 = 198.2 ± 8.8, Rag1 + Rag2 = 170.7 ± 14.9) or seed treatment (no ST = 168.5 ± 10.8, +ST = 182.0 ± 10.7) (ST × Line × Predator, *df*_3,46_, *F* = 0.604, *p* = 0.616; ST × Predator, *df*_1,46_, *F* = 0.657, *p* = 0.422; Line × Predator, *df*_3,46_, *F* = 1.694, *p* = 0.181; Predator, *df*_1,46_, *F* = 16.216, *p* < 0.001; ST × Line, *df*_3,46_, *F* = 1.774, *p* = 0.165; ST, *df*_1,46_, *F* = 1.075, *p* = 0.305; Line, *df*_3,46_, *F* = 1.356, *p* = 0.268). When only using the data from thrips-free plants, there was still no impact of the seed treatment on spider mite densities (ST × Predator, *df*_1,16_, *F* = 0.555, *p* = 0.467; Predator, *df*_1,16_, *F* = 12.846, *p* = 0.002; ST, *df*_1,16_, *F* < 0.001, *p* = 0.995; Line not used in model). Soybean variety and the seed treatment did not impact the density of *A. fallacis* offspring within clip cages to which predators were added (mean per cage ± SEM = 8.9 ± 1.1) (ST × Line, *df*_3,19_, *F* = 0.490, *p* = 0.693; ST, *df*_1,19_, *F* = 0.322, *p* = 0.577; Line, *df*_3,19_, *F* = 1.369, *p* = 0.282).

## 4. Discussion

In soybean production systems in the Midwest, insecticidal neonicotinoid seed treatments are commonly used to manage soybean aphids [11,50] and there is interest in using lines with *Rag* resistance genes [6,16]. However, both tactics can potentially affect non-target arthropods. We investigated how short-term reproduction of twospotted spider mites differed on soybean lines with and without *Rag1* and *Rag2* resistance genes and a thiamethoxam seed treatment in the presence of a specialist predator (phytoseiid mite, *A. fallacis*), while accounting for the presence of an opportunistic omnivore (thrips, *F. occidentalis*).

Reproduction of twospotted spider mites can depend on soybean cultivar [28,51]. However, within the limitations of our experiments, we did not find any impact of either *Rag* resistance gene on any of the non-target arthropods. Soybean aphid feeding induces changes in host plants that may impact other herbivores [52], and so in addition to the short duration of the experiments, it is important to note that we used plants that had not been exposed to this target pest. The amino acid composition of leaf tissue from soybean lines with *Rag1* genes is different than that of related plants lacking these genes [53]. Even so, consuming plant tissue from soybean lines with *Rag* genes did not alter fitness of western corn rootworm larvae (*Diabrotica virgifera virgifera* LeConte) [54], and other studies [22,55] concluded that the presence of *Rag1* genes had little impact on feeding (preference or defoliation) of several soybean pests within the Coleoptera and Lepidoptera, although in the latter study the authors documented reduced caterpillar growth rates. In contrast, Rich and Koch [23] found that brown marmorated stink bugs [*Halyomorpha halys* (Stål)] preferred soybeans with *Rag1*, and had lower levels of mortality when reared on pods from *Rag1* plants.

There is substantial literature on how pesticides, including sublethal doses, can impact arthropod biology and behavior and contribute to spider mite outbreaks in agroecosystems [1,4,41,56,57,58,59]. It is highly likely that in our experiments the systemic insecticide was present in plant tissue, especially because Magalhaes et al. [10] demonstrated that when soybean seeds were treated with the same rate of thiamethoxam (Cruiser^®^ 5FS, 50 g AI per 100 kg seed), the chemical was still present at detectable limits (3.5–9.6 ng/g leaf) in older and newer leaf tissue 40 days after planting, and was still negatively impacting the target pest (i.e., soybean aphid).

Spider mites (Tetranychidae) are not target pests of neonicotinoid insecticides [13,60]. However, there have been reports of increased problems with spider mites on plants treated with these chemicals [32] and so information on how neonicotinoids impact their densities or aspects of their biology related to population growth (e.g., mortality, fecundity, longevity, etc.) has been of considerable interest. In our study, the effect of the seed treatment on spider mite densities appeared to depend on the spatial scale of the experiment, with no impact when mites were confined to clip cages, but higher densities on seed treated whole soybean plants. Several researchers have found minimal or negative effects of neonicotinoids on spider mite density or biology in controlled lab or greenhouse experiments (e.g., leaf disc, detached leaf, clip cage or sticky ring on plant, whole plant) in the absence of predators [32,37,45,61,62,63,64]. In contrast, others have reported positive effects of these chemicals on spider mites in controlled experiments in both the absence [34,35,36,37,47,65,66] and presence of predators [43,63], and also in the field [32,33,34,37].

Collectively, the studies mentioned in the previous paragraph involve multiple types of neonicotinoid insecticides, different formulations and rates (and thus different ways in which the mites were exposed to the insecticides), multiple types of host plants, and several tetranychid species and strains. Compared to experiments using foliar sprays, soil granules, or soil drenches, there is less information in the literature on the effects of neonicotinoid seed treatments, especially thiamethoxam, on spider mites. Several non-mutually exclusive mechanisms that have been proposed to explain increased spider mite populations on neonicotinoid treated plants are that the chemical reduces interspecific competition [61,62], alters the mite reproductive physiology (i.e., hormesis, hormoligosis) or other life history parameters associated with population growth [47,65], increases the suitability of the plant as a host by altering plant nutrition and/or defense [34,35], or negatively affects natural enemy biology and behavior [32]. These mechanisms are similar to those previously proposed for explaining spider mite outbreaks following the application of other types of insecticides [59].

Neonicotinoids are known to negatively impact the biology and behavior of several types of natural enemies [34,39,40]. With regard to phytoseiid mites, some research has shown that these chemicals were not directly toxic [42,43,44,67] and others have found negative effects on survival, either by direct exposure or via residues or contaminated prey [38,68]. Adverse effects of consuming neonicotinoid-tainted spider mites have been noted for several predators, and include: higher mortality, reduced mobility, lower feeding rates, and/or reduced fecundity [34,42,43]. There is also evidence that neonicotinoids alter aspects of predator life history (e.g., fecundity), movement, and prey consumption [42,43,44,63,67]. In our study, *A. fallacis* reproduction and the suppression of spider mites was not adversely affected on seed treated plants. However, the arthropods were confined in a limited area and spider mite prey had only been feeding on the plant for 3 days prior to the predators being added, which may not have been long enough for any negative effects of consuming tainted prey to become apparent.

In the clip cage experiment, the seed treatment altered the relationship between the spider mites and an opportunistic omnivore (i.e., thrips) that colonized the experimental plants. On non-seed treated plants, there was a negative relationship between the densities of thrips and spider mites, perhaps indicating that thrips were consuming spider mites. On seed treated plants, there was not a significant relationship between the two arthropods, which may indicate that thrips were avoiding preying on spider mites. However, contrary to our expectations, spider mite densities were not higher on seed treated plants. We did not assess plant feeding by thrips, and so one possibility is that on seed treated plants thrips primarily consumed plant material, thus having an indirect negative effect on spider mites via reduced plant quality.

In addition to consuming spider mite immatures [46,69], some research indicates that thrips are attracted to spider mite infested plants [70], engage in behavior associated with searching for prey when exposed to chemicals extracted from spider mite webbing [71], and play an important role in regulating spider mite populations [72]. However, the degree to which thrips consume spider mites appears to depend on the growth stage and condition of the host plant, with thrips less attracted to spider mites when plants are flowering [69,73], thrips eating more spider mite eggs as plant quality decreases [74], and thrips eating fewer eggs laid by mites feeding on plants where induced plant resistance was triggered [75].

## 5. Conclusions

Understanding how pest control tactics impact non-target organisms, especially other herbivores and their biological control agents, is necessary to develop effective, sustainable IPM programs. In this study, the presence of *Rag1* and *Rag2* soybean aphid resistance genes did not impact the density of any non-target organism, lending support to the use of this technology for soybean aphid management. In contrast, spider mite densities were higher and correlations between densities of spider mites and an opportunistic early season predator (i.e., thrips) altered on soybeans with a neonicotinoid seed treatment, although these results were context-dependent. This research adds to the discussion about the compatibility of neonicotinoid insecticides within IPM programs and highlights the importance of considering the effects of management tactics on organisms other than focal pests.
